# Supplementary material for: A Tissue Paper/Hydrogel Composite Light-Responsive Biomimetic Actuator Fabricated by In Situ Polymerization
Source: Polymers (Basel). 2022 Dec 13;14(24):5454. doi: 10.3390/polym14245454 (PMC9785941; doi:10.3390/polym14245454)
Supplement: Supplementary file 1 [file polymers-14-05454-s001.zip › Revised Supporting information.pdf]

Supplementary Information for:

A Tissue Paper/Hydrogel Composite Light-Responsive Biomimetic Actuator Fabricated by In-Situ Polymerization

Qijun Wu [1,#], Chao Ma [2,#], Lian Chen [1], Ye Sun [2], Xianshuo Wei [1], Chunxin Ma [2,4*], Hongliang Zhao[4], Xiuling Yang [1], Xiaofan Ma [1], Chunmei Zhang [3,*] and Gaigai Duan [1,*]

[1] Jiangsu Co-Innovation Center of Efficient Processing and Utilization of Forest Resources, International Innovation Center for Forest Chemicals and Materials, College of Materials Science and Engineering, Nanjing Forestry University, Nanjing 210037, China

[2] State Key Laboratory of Marine Resource Utilization in South China Sea, Hainan University, Haikou 570228, China

[3] Institute of Materials Science and Devices, School of Materials Science and Engineering, Suzhou University of Science and Technology, Suzhou 215009, China

[4] Key Laboratory of quality safe evaluation and research of degradable material for State Market Regulation, Products Quality Supervision and Testing Institute of Hainan Province, Haikou 570203, China

[*] Corresponding author: machunxin@hainanu.edu.cn (C. Ma); cmzhang@usts.edu.cn (C. Zhang); duangaigai@njfu.edu.cn (G. Duan).

[#] These authors contributed equally to this work.

Supplementary Figures

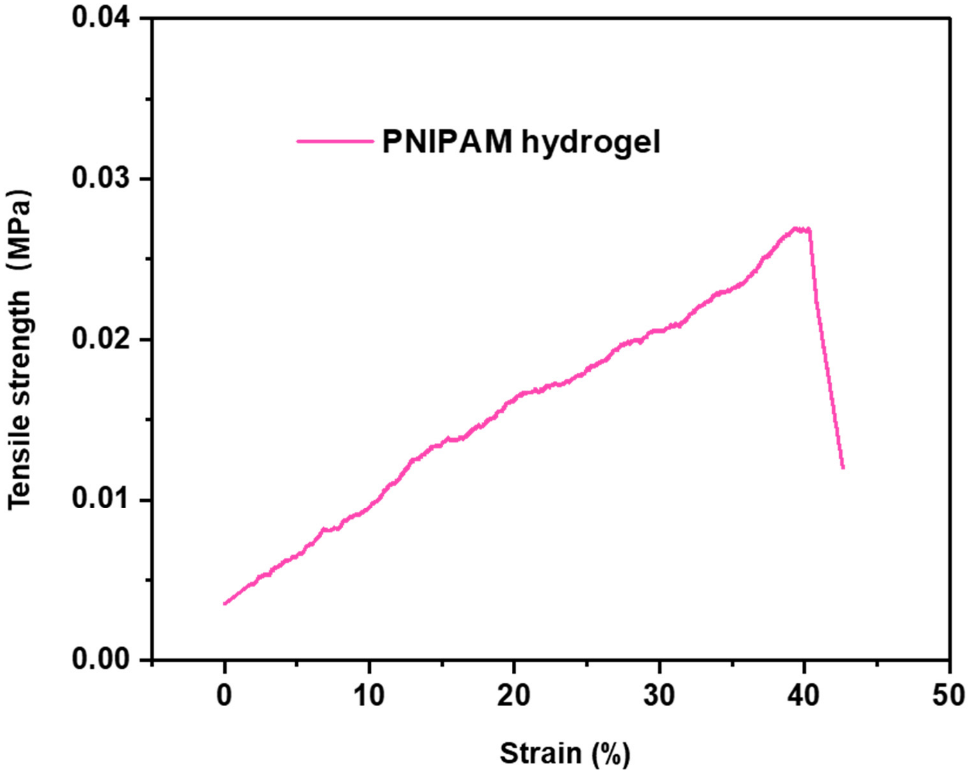

Figure S1. Mechanical property of the PNIPAM hydrogel.

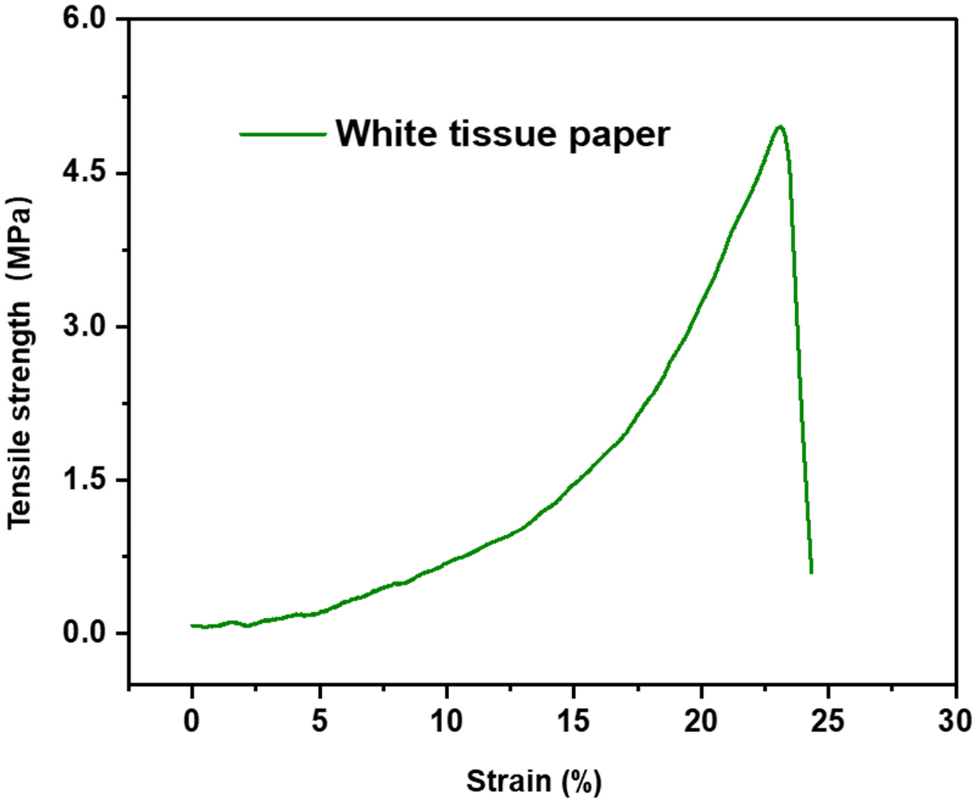

Figure S2. Mechanical property of the original white tissue paper.

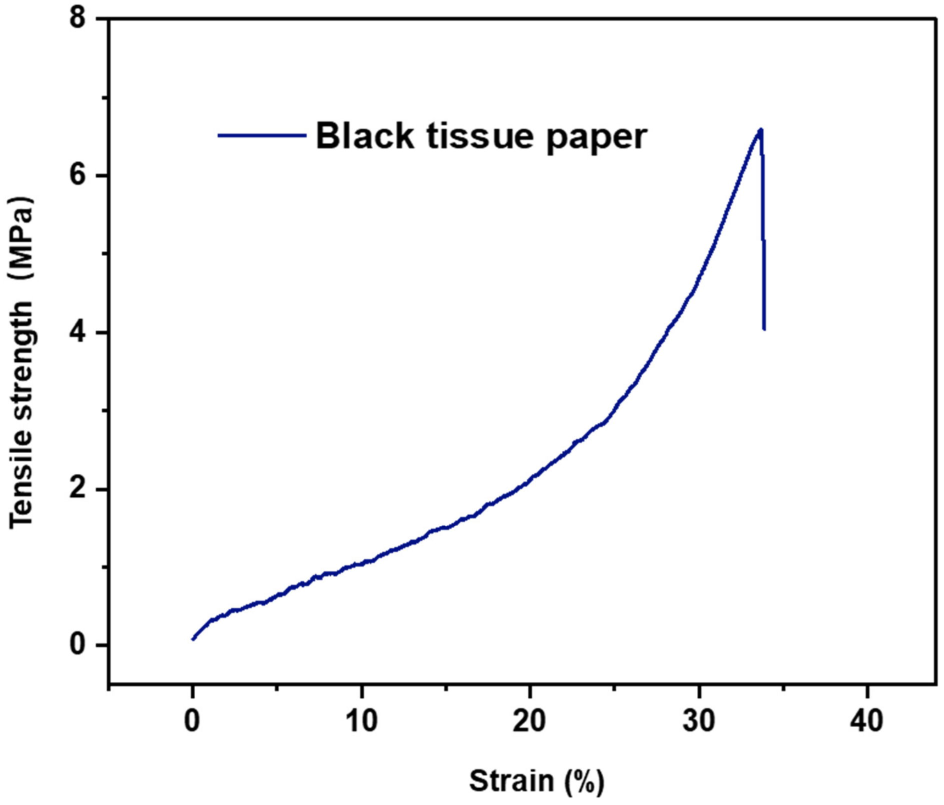

Figure S3. Mechanical property of the original black tissue paper dyed by the printer.

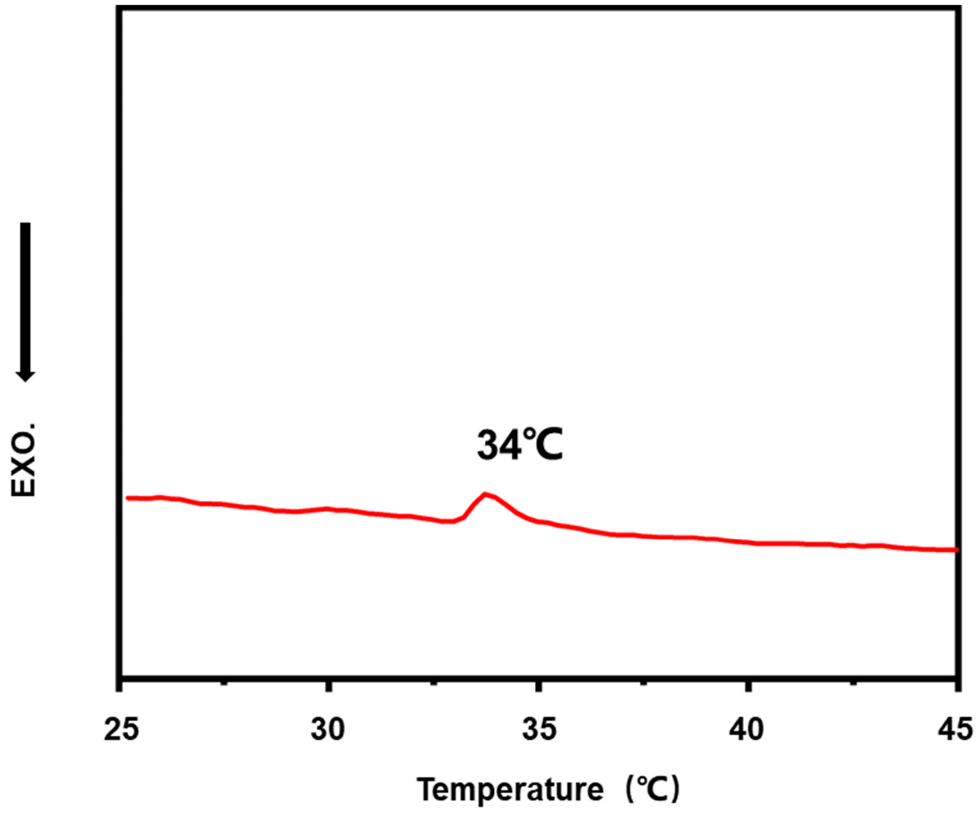

Figure S4. The DSC curve of the PNIPAM hydrogel.

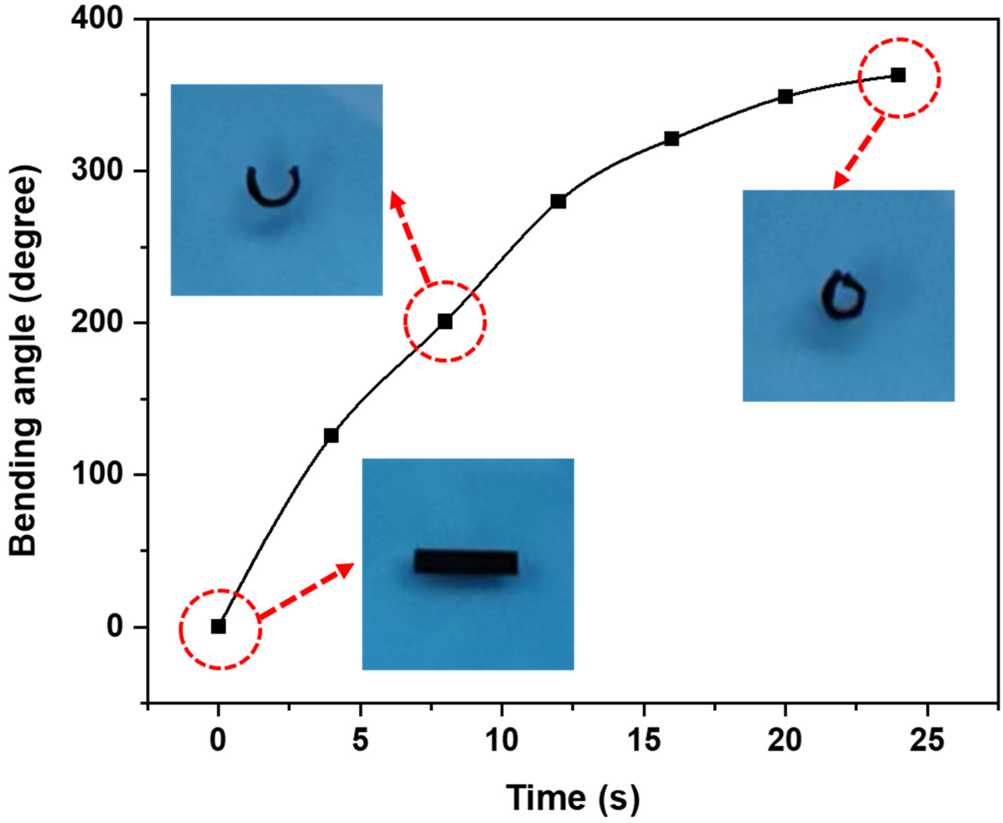

Figure S5. The bending process of the tissue paper/hydrogel composite actuator under a 45 °C warm water.

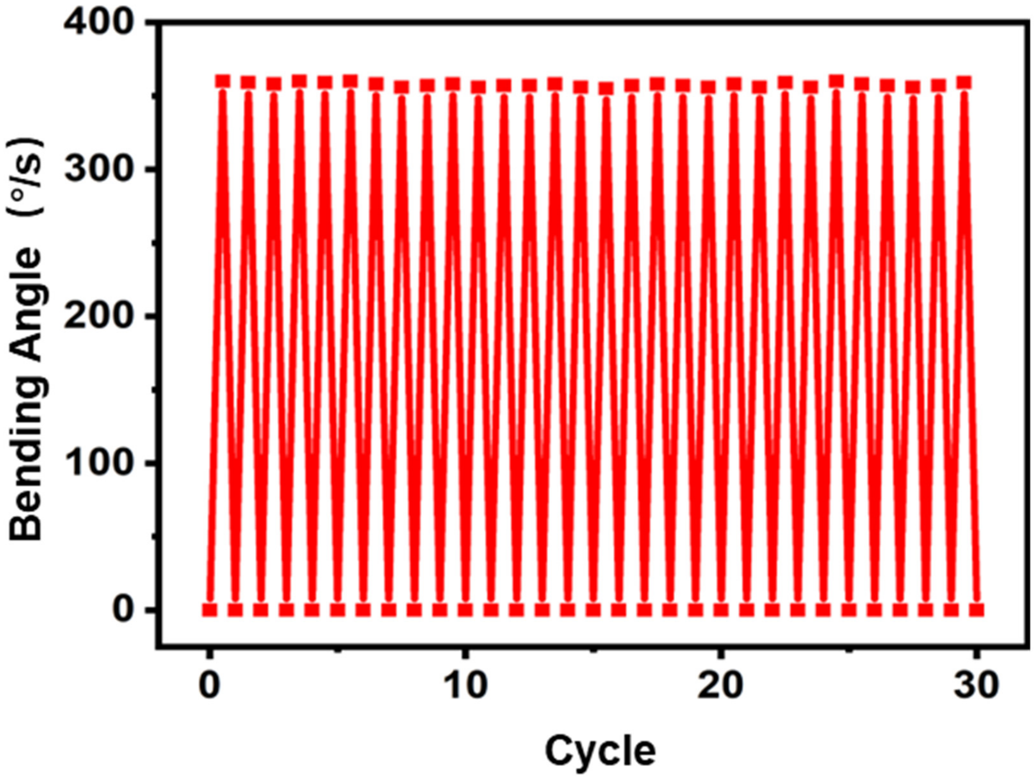

Figure S6. The bending-recovering cycle performance test of the tissue paper/PNIPAM hydrogel composite actuator.

Supplementary Movies

Movie S1. Bending-recovering process of the composite actuator under an area irradiation (with 3.5 w/cm^2 intensity of an 808 nm laser lamp).

Movie S2. Folding-recovering process of the composite actuator under a point irradiation (with a 0.2 cm of spot diameter and 2 W of power by an 808 nm laser lamp).

Movie S3. The remotely-controlled light-responsive grabbing behavior of the bio-mimetic "paw" based on the composite actuator under an area irradiation (with 3.5 W/cm^2 intensity of an 808 nm laser lamp).

Movie S4. The remotely-controlled light-responsive finger-folding performance of the bio-mimetic "hand" based on the composite actuator under a point irradiation (with a 0.2 cm of spot diameter and 2 W of power by an 808 nm laser lamp, 63.9 W/cm^2).
